# Validation of the GSP^®^/DELFIA^®^ Anti-SARS-CoV-2 IgG Kit Using Dried Blood Samples for High-Throughput Serosurveillance and Standardized Quantitative Measurement of Anti-Spike S1 IgG Antibody Responses Post-Vaccination

**DOI:** 10.3390/vaccines10040514

**Published:** 2022-03-26

**Authors:** Ilaria Cicalini, Piero Del Boccio, Mirco Zucchelli, Claudia Rossi, Luca Natale, Gianmaria Demattia, Domenico De Bellis, Verena Damiani, Maria Lucia Tommolini, Erika Pizzinato, Alberto Frisco, Sara Verrocchio, Ines Bucci, Liborio Stuppia, Vincenzo De Laurenzi, Damiana Pieragostino

**Affiliations:** 1 Center for Advanced Studies and Technology (CAST), “G. d’Annunzio” University of Chieti-Pescara, 66100 Chieti, Italy; ilaria.cicalini@unich.it (I.C.); piero.delboccio@unich.it (P.D.B.); m.zucchelli@unich.it (M.Z.); claudia.rossi@unich.it (C.R.); lucanatale1989@gmail.com (L.N.); gianmaria.d@hotmail.it (G.D.); domenico.debellis@unich.it (D.D.B.); verena.damiani@unich.it (V.D.); luciamaria9@hotmail.it (M.L.T.); rikerika1396@hotmail.com (E.P.); albertofrisco@live.it (A.F.); sara.verrocchio@unich.it (S.V.); ines.bucci@unich.it (I.B.); stuppia@unich.it (L.S.); delaurenzi@unich.it (V.D.L.); 2 Department of Innovative Technologies in Medicine and Dentistry, “G. d’Annunzio” University of Chieti-Pescara, 66100 Chieti, Italy; 3 Department of Pharmacy, “G. d’Annunzio” University of Chieti-Pescara, 66100 Chieti, Italy; 4 Department of Psychological, Health and Territory Sciences, School of Medicine and Health Sciences, “G. d’Annunzio” University of Chieti-Pescara, 66100 Chieti, Italy; 5 Department of Medicine and Aging Science, “G. d’Annunzio” University of Chieti-Pescara, 66100 Chieti, Italy

**Keywords:** anti-SARS-CoV-2 antibody test, anti-SARS-CoV-2 vaccines, serological quantitative test, DBS

## Abstract

Severe acute respiratory syndrome coronavirus-2 (SARS-CoV-2) has caused a major global public health crisis. In response, researchers and pharmaceutical companies worked together for the rapid development of vaccines to reduce the morbidity and mortality associated with viral infection. Monitoring host immunity following virus infection and/or vaccination is essential to guide vaccination intervention policy. Humoral immune response to vaccination can be assessed with serologic testing, and indeed, many serological immunoassays are now in use. However, these many different assays make the standardization of test results difficult. Moreover, most published serological tests require venous blood sampling, which makes testing large numbers of people complex and costly. Here, we validate the GSP^®^/DELFIA^®^ Anti-SARS-CoV-2 IgG kit using dried blood samples for high-throughput serosurveillance using standard quantitative measurements of anti-spike S1 IgG antibody concentrations. We then apply our validated assay to compare post-vaccination anti-SARS-CoV-2 S1 IgG levels from subjects who received a double dose of the AZD1222 vaccine with those vaccinated with a heterologous strategy, demonstrating how this assay is suitable for large-scale screening to achieve a clearer population immune picture.

## 1. Introduction

Severe acute respiratory syndrome coronavirus-2 (SARS-CoV-2) caused the COVID-19 pandemic in 2019. Although many control measures such as the use of face masks, distancing and lockdowns have been adopted by all the countries of the world, COVID-19 has spread everywhere. Therefore, vaccines have been developed and implemented globally to reduce the morbidity and mortality associated with SARS-CoV-2 [1]. SARS-CoV-2 binds the host angiotensin-converting enzyme 2 (ACE2) by a multi-step process involving three separate S protein cleavage events. In particular, the most important protein domains are S1 and S2, which are involved in ACE2 binding, membrane fusion and cell entry. Therefore, most vaccines have been designed with the goal of inducing a high number of anti-S1 antibodies, with the aim of reducing cell invasion and viral replication. Humoral immune response to infection, as well as to vaccination, can be assessed by measuring circulating antibodies. Thus, many serological immunoassays have been developed for this purpose. These tests can be used for the detection of anti-SARS-CoV-2 IgM antibodies, already measurable in the blood a few days after the onset of infection (and/or vaccination), and anti-SARS-CoV-2 IgG antibodies, which become detectable above all 7–10 days after infection [2]. The latter have become extremely important in assessing the response to SARS-CoV-2 vaccination. Protocols for the SARS-CoV-2 antibody assay using serum or plasma obtained via venipuncture are well established in a clinical setting. However, this assay is not suitable for large-scale studies due to the logistic constraints related to blood taking, storage, processing and transportation. On the other hand, capillary blood collection on filter paper cards after finger pricking, called dried blood spots (DBS), can act as a valid and advantageous alternative, considering the low invasiveness of the collection method, which can also be used for self-collection, and the simple and low-cost shipping and storage [3]. A DBS-based serological assay measuring IgG antibodies to S1 spike (PerkinElmer) was already used in our previous works to highlight anti-SARS-CoV-2 antibody titration during the vaccination campaign in Italy. Our data showed that such a test can monitor antibody fluctuation following vaccination and that large-scale monitoring is possible [4,5]. Moreover, we demonstrated that DBS IgG monitoring can be applied to newborns by using the same DBS used for neonatal screening (mandatory in Italy) [6]. The GSP^®^/DELFIA^®^ Anti-SARS-CoV-2 IgG test (PerkinElmer) used in our previous studies calculates a ratio of the fluorescence of the sample over the calibrator as reported in the manufacturing kit protocol, obtaining results not comparable with values measured using other methods and leading to doubts and difficulties surrounding result interpretation at large.

In fact, the use of serological tests in clinical practice as a tool for monitoring the vaccination campaign presents a series of problems and limitations, mainly linked to the difficulty in comparing the results of IgG levels obtained by different laboratories using different assays. Serological tests can in fact be based on the use of different antigens, for example: the SARS-CoV-2 nucleocapsid (N) antigen, the whole virus lysate antigen, the S1 domain antigen or the Spike S1-RBD domain [7]. In particular, spike antigens are optimal in monitoring the efficacy of vaccines, while nucleocapsid antigens are particularly suitable for monitoring natural viral infections.

Moreover, it should be considered that antibody levels can be expressed in many units of measurement, such as arbitrary units (a.u.), binding antibody units (BAU), U/mL, µg/mL, pg/mL or a ratio of the fluorescence of the sample over the calibrator [5,7]. All these methodological differences make result comparison very difficult, and therefore inhibit the application of a population-wide survey [8]. Thus, in this work, we validated the GSP^®^/DELFIA^®^ Anti-SARS-CoV-2 IgG test for the measurement of IgG antibodies to S1 spike via DBS from the perspectives of linearity, precision, accuracy and stability over time, expressing IgG levels in standard comparable units (ng/mL) through a method suitable for large-scale application. 

We then applied our validated method to compare the anti-SARS-CoV-2 IgG levels from subjects who received a double dose of the AZD1222 vaccine with those who were vaccinated with a heterologous strategy.

## 2. Materials and Methods

### 2.1. Anti-S1 Spike IgG Immunoassay

Capillary blood samples were collected on filter paper cards by using finger-prick lancets. The blood sample was dried for at least one hour and stored at room temperature with a desiccant dehumidifier. From the entire drop of dried blood (which represents a volume of about 25 µL), a spot with a 3.2 mm DBS disk was taken for analysis, using the automatic Puncher (Panthera-Puncher, PerkinElmer^®^, Turku, Finland). This sample collection system ensured that the same amount of sample was taken and analyzed for each test.

IgG antibodies to SARS-CoV-2 spike S1 were measured via a fully automated solid phase DELFIA (time-resolved fluorescence) immunoassay, by using a GSP^®^/DELFIA^®^ Anti-SARS-CoV-2 IgG kit on a GSP instrument (PerkinElmer^®^, Turku, Finland). The results obtained in terms of fluorescence were normalized to the fluorescence of a sample of known concentration called the calibrator (300 ng/mL). For this reason, results are indicated as a ratio, without a unit of measurement. The ratio values of internal quality control samples (QCs) were used to evaluate the analytical performance of the method, described in more detail in the following paragraph. The positive limit of the test was set using 1.2 as a cut-off value expressed as a ratio, which corresponds to 411.5 ng/mL. This positivity cut-off value was established using a series of samples consisting of one thousand two hundred and sixty-nine subjects, including subjects who had SARS-CoV-2 infection and healthy subjects, from November 2020 to January 2021, and therefore without having received the vaccine administration, as already described and published in our previous work [5]. 

### 2.2. Assay Performance Characteristics

With the aim of making this test a quantitative dosage, we performed many analytical experiments assessing the test performance in terms of linearity, precision, accuracy and stability over time. DBS QC samples with increasing concentrations of IgG anti-SARS-CoV-2 from 0 to 10,000 ng/mL were obtained from PerkinElmer^®^, Turku, Finland. Linearity was defined as a deviation of 15% from the assigned calibrator values, except for the lowest limit of quantification (LOQ), for which a deviation of ≤20% was accepted. The precision and accuracy were evaluated intra- and inter-day using the QC samples described above. For intra-assay precision, a sequence of three QC replicates was analyzed in a single lot, while three QC replicates on five consecutive days were used for inter-assay studies. The intra and inter-test precisions were expressed as CV% and standard deviation (SD) from the assigned values. The limit of detection (LOD) and LOQ were determined by analyzing a sequence of 28 blank samples, calculating the average of measurements plus SD*3 and SD*10. The stability of the samples was evaluated by analyzing 5 samples stored at room temperature, at +4 °C and at −20 °C for up to 31 days.

### 2.3. Patient Enrolled and Sample Collection

DBS samples were collected at the Center for Studies and Advanced Technologies (CAST), “G. d’Annunzio” of Chieti-Pescara, Italy. A total of 113 subjects underwent full vaccination with two doses of the Astra Zeneca vaccine AZD1222 (AZ), while 58 subjects underwent heterologous vaccination: 44 subjects received Pfizer BNT162b2 (AZ + Pf) and 14 Spikevax (Moderna) for the second dose. Samples were collected following the approval of the Ethics Committee No. 19 of 9 September 2021. The study was conducted according to the guidelines of the Declaration of Helsinki, and informed consent forms were obtained from the enrolled subjects.

### 2.4. Statistical Analysis

A *t*-test and Kruskal–Wallis test with Dunn’s multiple comparisons post-test were performed to evaluate the significance of the measurements. A *p*-value of < 0.05 was set as a significant value. Linear regression with 95% confidence intervals was used for line fitting experiments, calculating R^2^ and the related linear equation by using GraphPad Prism 7.0. 

## 3. Results

### 3.1. Linearity Repeatability and Accuracy and Stability over Time

Method linearity was established by analyzing QC samples from 0 to 1000 ng/mL (0, 100, 200, 300 and 500 ng/mL) and levels from 500 to 10,000 ng/mL (500, 1000, 3000 and 10,000 ng/mL) five times in different analytical runs. Considering the wide concentration range, we decided to test method linearity, dividing the fluorescence results into two calibration ranges, as described in Table 1 and Figure S1. 

Table 1 shows the investigated concentration ranges, the calculated calibration functions, and the corresponding coefficient of determination (R^2^). The R^2^ values were >0.98 over their concentration ranges, fitting well in the linear regression model.

The repeatability was established by analyzing intra- and inter-assay variability at seven levels of QC from 100 to 10,000 ng/mL. In Table 2, we report, for each QC level, the nominal concentration, the mean of the measured concentration, the SD and the CV%. The intra- and inter-assay CVs from the assigned values were ≤6.83% and ≤14.80%, respectively, for all QC levels. The accuracy of the calculated values was established through the calculation of bias% from seven QCs (100, 200, 300, 500, 1000, 3000 and 10,000 ng/mL). The only value that appeared to have a bias % outside the accepted range is the 100 ng/mL level, for which an average accuracy of 41.2% was calculated. The other QCs had less than 20% bias, as shown in Table 2.

**Figure 1:**
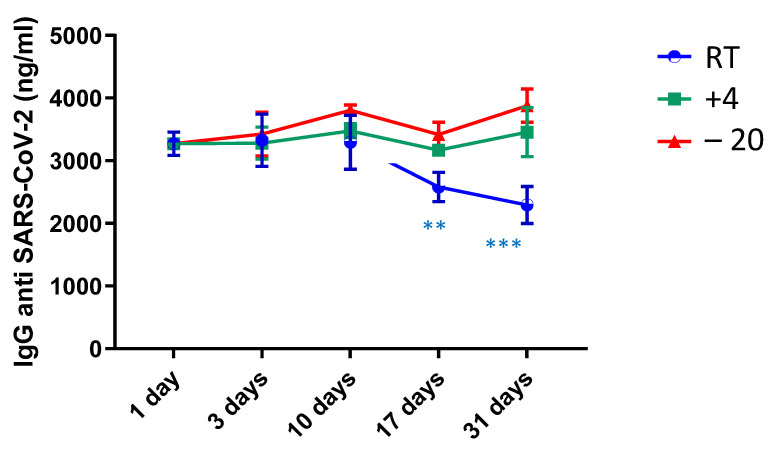
IgG anti SARS-CoV-2 measured up to 31 days after the sample collection at room temperature (RT), at +4 °C, at −20 °C. ** means *p*-value at *t*-test < 0.001, *** means *p*-value at *t*-test < 0.0001.

The LOD and LOQ were determined by analyzing 28 DBS samples with no IgG anti-SARS-CoV-2 (blank matrix) and calculating the mean and the SD of the measurements. The LOD and LOQ were established to be 7.78 ng/mL and 44.15 ng/mL, respectively. Stability tests were carried out, keeping aliquots (*n* = 5) of a selected DBS sample at three different temperature conditions: room temperature (RT), +4 °C and −20 °C. Samples were analyzed on the first day of collection and after 3, 10, 17 and 31 days of storage, as described. As shown in Figure 1, no significant reduction in IgG concentration was found by storing the DBS sample at +4 and −20 °C, while a significant drop in IgG was observed by storing the sample at RT (about 25 °C) after 10 days.

### 3.2. Evaluation of IgG Anti-SARS-CoV-2 Levels in Subjects Undergoing Heterologous Vaccination

**Figure 2:**
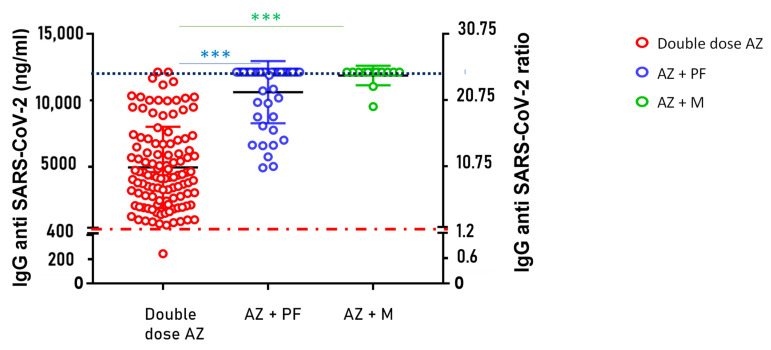
IgG anti-SARS-CoV-2 levels, expressed as ng/mL (Left *Y*-axis) and expressed as ratio (Right *Y*-axis), in 113 subjects up to 30 days after the second dose of AZ vaccine in red, 44 subjects up to 30 days after the mix of AZ and PF vaccines in blue and 14 subjects up to 30 days after the mix of AZ and Moderna vaccines. All the IgG values measured above the upper limit of quantification of the method were expressed as 12,125 ng/mL (ratio of 25), highlighted by the dashed blue line. The red dashed line represents the cutoff of positivity set at 411.5 ng/mL. *** means *p*-value at Kruskal–Wallis test and Dunn’s multiple comparison test <0.0001.

The quantitative evaluation of our method in the monitoring of IgG anti-SARS-CoV-2 levels from DBS samples was applied in a post-vaccine study. We analyzed 113 subjects who received a double dose of the AZD1222 vaccine (AZ), 44 subjects who received the first dose of AZD1222 and the second dose of BNT162b2 (AZ + Pf), and finally, 14 subjects who received the AZD1222 vaccine for the first dose and the Spikevax (Moderna) vaccine for the second dose (AZ + M). All samples were collected 30 days after the administration of the last dose of the vaccine, and IgG anti-SARS-CoV-2 levels were expressed in ng/mL. The limit of positivity was set to the value of 411.5 ng/mL, which corresponded to 1.2 in terms of the fluorescence ratio. As shown in Figure 2, a significant increase in SARS-CoV-2 IgG antibody titers was observed in the heterologous vaccination strategy after the Kruskal–Wallis test (*p*-value < 0.001). A significant increase in antibody titer was observed in the comparison between the double dose of AZ and the mix of AZ and PF (*p*-value at Dunn’s multiple comparisons test < 0.0001), as well as between double dose of AZ and the mix of AZ and Moderna (*p*-value at Dunn’s multiple comparisons test < 0.0001). However, no significant difference was observed comparing the two heterologous vaccination strategies (AZ + PF and AZ + M).

After a complete cycle of anti-SARS-CoV-2 vaccination, IgG levels in the blood reached values much higher than the maximum limit of linearity obtained by our method at 12,125 ng/mL. This limit is 25 in terms of fluorescence ratio, as also reported by the commercialized kit (see Figure 2). 

To prove the linearity of the method in quantifying IgG values above 12,125 ng/mL (ratio of 25), we analyzed DBS taken from successive dilutions of blood taken from a volunteer who had received three doses of the BNT162b2 vaccine and after SARS-CoV-2 infection, and who therefore showed very high levels of IgG (ratio 80). Dilutions were made using antibody-negative blood from a volunteer without vaccination coverage (ratio 0.33).

**Figure 3:**
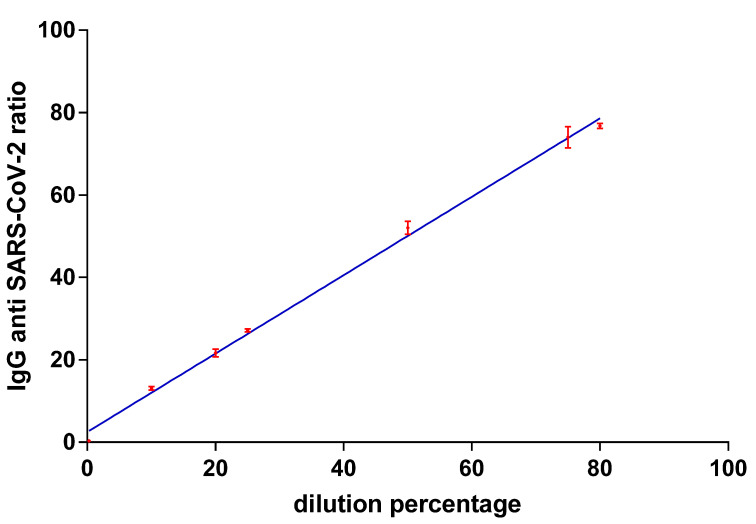
Linear regression between anti-SARS-CoV-2 IgG levels, expressed as a ratio, and the dilution percentage starting from a DBS sample from a subject with three doses of BNT162b2 vaccine and with previous SARS-CoV-2 infection (*n* = 3). Linear regression calculated shows R^2^ = 0.99, slope of the line equal to 0.95 (*p*-value < 0.0001).

As reported in Figure 3, there is a linear correlation between the dilution percentages and the IgG values in terms of ratios up to values equal to 80, observing an R^2^ equal to 0.99 and a slope coefficient of the curve equal to 0.95 (*p*-value of linear regression <0.0001). This would allow us a reasonable quantification up to 39,625 ng/mL (ratio 80), useful for discriminating the immune response in subjects who have had the booster vaccine dose from 7 days and up to months later (see Figure S2). Above this value, the trend is no longer linear, and for this reason, the data could not be accurately quantified (data not shown).

## 4. Discussion

The progression of the pandemic worldwide, in relation to the development of variants and the need for access to new vaccine boosters, has required urgent action by governments to cope with the rapid increase in infection in the population. One of the main objectives was to determine the length of time that should be allotted after the last injection before administering the booster dose of the vaccine, which, implicitly, is meanly correlated to antibody levels. In fact, vaccine-induced immune responses are often multifaceted, but single components such as antibody responses may correlate with the level of protection. Therefore, there is an increasing need for a test that can be used for serosurveillance, regulating access to vaccine doses more properly.

In fact, most of the currently accepted protection-related criteria are based on antibody measurements since they are often much easier to measure than cellular responses and are therefore more clinically useful. For instance, it is known that, for smallpox (a virus that infects the mucosae), antibody provides the best correlation of immunity to infection after vaccination, but cellular responses, particularly CD8+ cells, influence the severity of the disease if infection occurs despite antibodies [9]. Moreover, Earle et al. show a robust, positive correlation both between neutralizing titer and efficacy and between binding antibody titer and efficacy in COVID-19 vaccination. However, the study was limited by the geographically diverse populations subject to different forces of infection and circulating variants and the use of different endpoints, assays, convalescent sera panels and manufacturing platforms [10]. Recently, Khoury et al. highlighted the decay of the neutralization titer over the first 250 days after immunization and, consequently, predicted that a significant loss in protection from SARS-CoV-2 infection will occur, although protection from severe disease should be largely retained [11]. Therefore, the use of the serological test seems to be a promising tool for scheduling access to the vaccine booster and to study antibody fluctuations after vaccination/infection. Nonetheless, the available serological tests are heterogeneous in terms of methods, design, antibodies class detected, performance and result reporting [8]. Therefore, the standardization and interchangeability of results have been not reached so far, and, for the most part, the identification of a threshold value indicating the immune protection or the need for a booster vaccine dose is still lacking. Furthermore, many serological tests require venous sampling and laborious processing (centrifugation, analysis and storage), making testing large numbers of people extremely complicated and expensive [10]. The DBS, on the other hand, boasts all the necessary characteristics to become the reference test for serological analysis on a large population, as also described by Khan et al. [12]. Moreover, published studies have compared DBS tests with related serum samples, demonstrating a good correlation between the two biological fluids [13,14], finding for some tests a decreased sensitivity of the DBS compared to immunoassays on plasma [15]. Our data demonstrate the feasibility of such serological test for the large-scale screening application from different points of view: (i) the spot sampling is stable for the antibody count, even for 10 days at room temperature, and months if suitably stored; (ii) intra-day and inter-day assay showed excellent repeatability; (iii) the quantitative range of application is wide, covering the levels of antibodies mainly represented in the population, from the negative condition (no vaccine, no disease) to people with three doses of vaccination. Moreover, the technology employed for such tests, routinely used in neonatal screening for the diagnosis of rare diseases, can process thousands of samples per day through a standardized (ng/mL) anti-SARS-CoV-2 IgG measurement. In fact, our data on homologous and heterologous AZ vaccination show how the DBS methods can distinguish different vaccination responses and can help in understanding the right time at which a vaccination booster should be required. Our previous data indicate that it is possible to follow IgG fluctuation after vaccination even in newborns, and moreover, the quantitative results reinforce the idea that this test may be applied to different laboratories and may return an indicative limit value in accessing vaccination boost [4,5]. Moreover, the test could be useful to screen the pediatric population through a school-based survey to identify the many who have had an asymptomatic disease, reclassifying them in the vaccination campaign and reducing parent anxiety and reluctance towards vaccination. 

## 5. Conclusions

In conclusion, our work demonstrates how the high-throughput method for measuring anti-S1 antibodies against COVID-19 from DBS is suitable for quantitative applications since the titer is expressed in ng/mL, making the results standardized and demonstrating very good analytical and pre-analytical features. The high number of processable samples, the ease of sampling, the stability at room temperature and the very low waste produced also make it a technique applicable to population screening, including therein children and newborns, with the aim of having a population immunity picture against COVID-19 as a useful tool for serosurveillance and for making decisions in vaccination campaigns. 

## Figures and Tables

**Table 1:** Concentration ranges, calculated calibration functions and correlation coefficients (R^2^). b1 and b0 are mean values (*n* = 5).

Concentration Range (ng/mL)	Calibration Function Y = b1(±SD) x + b0(±SD) *n* = 5	R^2^ (±SD) *n* = 5
0–000	y = 0.0028(±0.00027) x + 0.048(±0.04)	0.99 (± 0.004)
500–10,000	y = 0.002(±0.00016) x + 0.75(±0.17)	0.99 (± 0.004)

**Table 2:** Measured concentration (ng/mL), standard deviation (SD) and CV% measured in *n* = 3 replicates intra-assay and *n* = 3 replicated for five consecutive days for inter-assay. Accuracy expressed as bias % and SD calculated in *n* = 5 replicates for each QC IgG level.

	Intra-Assay			Inter-Assay			Accuracy	
QC Levels (ng/mL)	Measured Concentration (ng/mL) *n* = 3	SD *n* = 3	CV%	Measured Concentration (ng/mL) *n* = 15	SD *n* = 15	CV%	Bias% *n* = 5	SD (ng/mL) *n* = 5
100	118.5	3.57	3.01	141.4	21.0	14.8	41.28	±12.08
200	218.6	7.14	3.26	190.3	22.8	12.0	−3.94	±2.48
300	298.3	8.98	3.01	288.6	38.0	13.2	−3.83	±4.37
500	568.6	34.1	5.99	453.6	60.1	13.2	−7.44	±6.44
1000	1097.1	75.0	6.83	1015.7	96.7	9.5	1.55	±1.51
3000	3193.3	42.5	1.3	3472.0	417.2	12.0	20.72	±4.48
10,000	10028.3	360.9	3.6	9271.7	732.3	7.9	−1.64	±1.54

## Data Availability

Data are contained within the article.

## Supplementary Materials

The following are available online at https://www.mdpi.com/article/10.3390/vaccines10040514/s1 Figure S1: Linear regression between anti-SARS-CoV-2 IgG levels, expressed as a ratio, and the nominal concentration, expressed as ng/mL between 0 and 1000 ng/mL (Panel A) and between 0 and 10,000 ng/mL (Panel B). Figure S2: IgG anti-SARS-CoV-2 levels, expressed as ng/mL (Left *Y*-axis) and expressed as ratio (Right *Y*-axis), in 113 subjects up to 30 days after the second dose of AZ vaccine in red, 44 subjects up to 30 days after the mix of AZ and PF vaccines in blue and 14 subjects up to 30 days after the mix of AZ and Moderna vaccines. All data are reported until our quantification upper limits, which correspond to 39,625 ng/mL (ratio 80), as highlighted by the blue dashed line. **** means *p*-value at Kruskal–Wallis test and Dunn’s multiple comparison test <0.0001.
