# Supplementary material for: Validation of the GSP®/DELFIA® Anti-SARS-CoV-2 IgG Kit Using Dried Blood Samples for High-Throughput Serosurveillance and Standardized Quantitative Measurement of Anti-Spike S1 IgG Antibody Responses Post-Vaccination
Source: Vaccines (Basel). 2022 Mar 26;10(4):514. doi: 10.3390/vaccines10040514 (PMC9028589; doi:10.3390/vaccines10040514)
Supplement: Supplementary file 1 [file vaccines-10-00514-s001.zip › vaccines-1631261-supplementary.pdf]

vaccines

Supplementary material

A

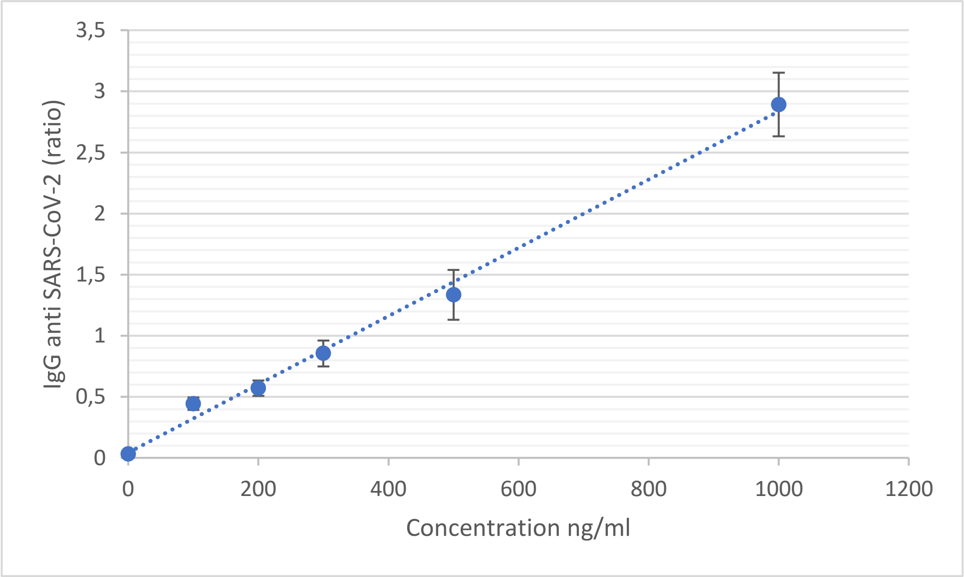

B

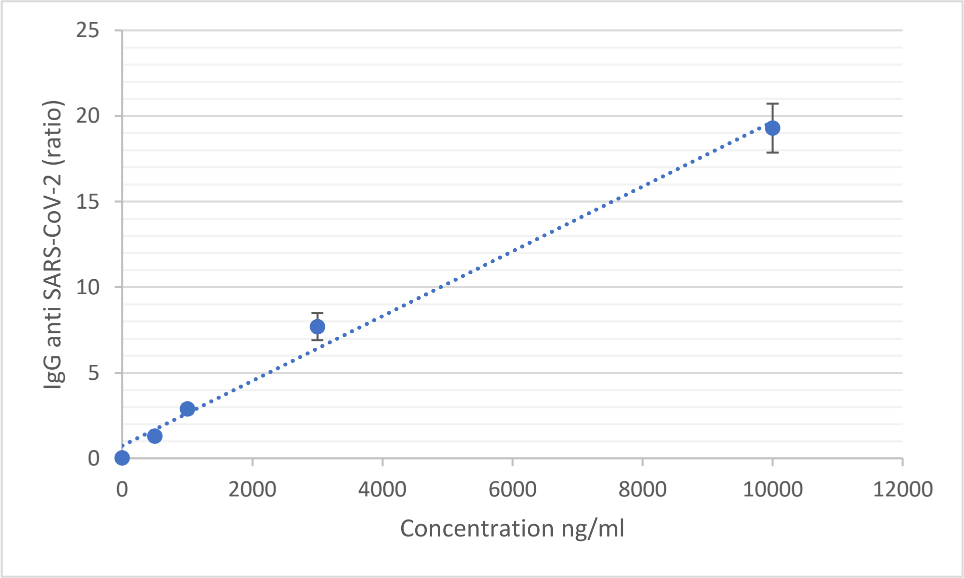

Figure S1: Linear regression between anti-SARS-CoV-2 IgG levels, expressed as a ratio, and the nominal concentration, expressed as ng/ml between 0 to 1000 ng/ml (Panel A) and between 0-10000 ng/ml (Panel B).

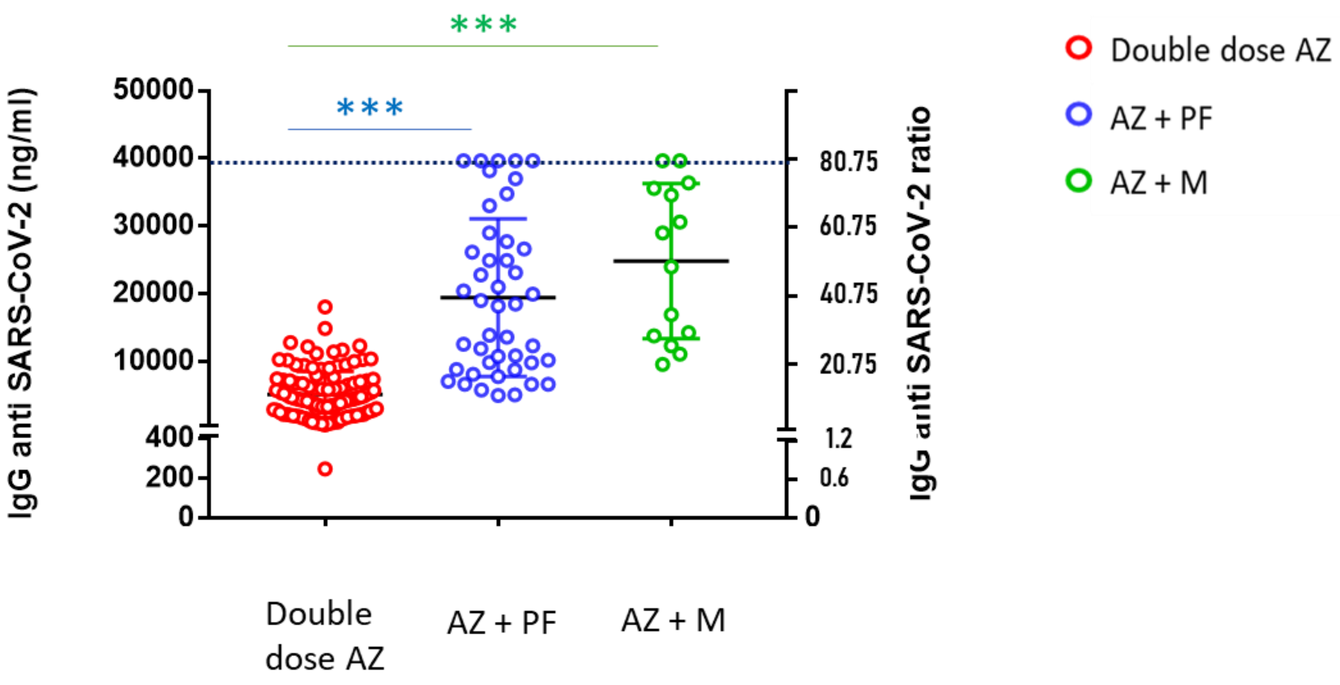

Figure S2: IgG anti-SARS-CoV-2 levels, expressed as ng/ml (Left Y axis) and expressed as ratio (Right Y axis), in 113 subjects up to 30 days after the second dose of AZ vaccine in red, 44 subjects up to 30 days after the mix of AZ and PF vaccines in blue, and 14 subjects up to 30 days after the mix of AZ and Moderna vaccines. All data are reported until our quantification upper limits, which correspond to 39625 ng/ml (ratio 80), as highlighted by the blue dashed line. *** means p-value at Kruskal Wallis test and Dunn's multiple comparison test <0.0001
